# A Comparative Study of Removal of Acid Red 27 by Adsorption on Four Different Chitosan Morphologies

**DOI:** 10.3390/polym16071019

**Published:** 2024-04-08

**Authors:** Hongli Wu, Jiaying Zhou, Sai Zhang, Ping Niu, Haoming Li, Zhongmin Liu, Ning Zhang, Chunhui Li, Liping Wang, Yudong Wang

**Affiliations:** 1 College of Textile and Light Industry, Inner Mongolia University of Technology, Hohhot 010081, China; wuhongli0321@126.com (H.W.); zhoujiaying202403@163.com (J.Z.); 2 College of Chemistry and Chemical Engineering, Dezhou University, Dezhou 253023, China; np68@sina.com (P.N.); liuzhongmin@dzu.com (Z.L.); znzwj@aliyun.com (N.Z.); 3 College of Textile and Clothing, Dezhou University, Dezhou 253023, China; zhangsai235@163.com; 4 College of Biological and Chemical Engineering, Guangxi University of Science & Technology, Liuzhou 545006, China; 19101248814@163.com

**Keywords:** chitosan, acid dye, structure, adsorption capacity and rate, crystallinity

## Abstract

To investigate the relationship between structures and adsorption properties, four different morphologies of chitosan, with hydrogel (CSH), aerogel (CSA), powder (CSP), and electrospinning nanofiber (CSEN) characteristics, were employed as adsorbents for the removal of Acid Red 27. The structures and morphologies of the four chitosan adsorbents were characterized with SEM, XRD, ATR-FTIR, and BET methods. The adsorption behaviors and mechanisms of the four chitosan adsorbents were comparatively studied. All adsorption behaviors exhibited a good fit with the pseudo-second-order kinetic model (R^2^ > 0.99) and Langmuir isotherm model (R^2^ > 0.99). Comparing the adsorption rates and the maximum adsorption capacities, the order was CSH > CSA > CSP > CSEN. The maximum adsorption capacities of CSH, CSA, CSP, and CSEN were 2732.2 (4.523), 676.7 (1.119), 534.8 (0.885), and 215.5 (0.357) mg/g (mmol/g) at 20 °C, respectively. The crystallinities of CSH, CSA, CSP, and CSEN were calculated as 0.41%, 6.97%, 8.76%, and 39.77%, respectively. The crystallinity of the four chitosan adsorbents was the main factor impacting the adsorption rates and adsorption capacities, compared with the specific surface area. With the decrease in crystallinity, the adsorption rates and capacities of the four chitosan adsorbents increased gradually under the same experimental conditions. CSH with a low crystallinity and large specific surface area resulted in the highest adsorption rate and capacity.

## 1. Introduction

Synthetic dyes are widely employed in the textile industry, and a significant number of them have carcinogenicity and mutagenicity [1]. The dye molecules in textile wastewater are stable for oxidants and microorganisms that pose challenges to elimination [2]. Consequently, various techniques have been developed to treat wastewater contaminated with dyes, including adsorption [3], membrane filtration [4,5], electrochemical treatment [6], advanced oxidation processes [7], enhanced biotechnology methods [8], and so on. Among them, adsorption has been a subject of extensive studies, due to its operational convenience and high efficiency, with a central focus on achieving high adsorption rates and capacities [9].

Chitosan is widely acknowledged as an eco-friendly and efficient adsorbent for dyeing wastewater. Different morphologies and structures of adsorbents based on chitosan were developed for dye removal, such as powder, bead, film, hydrogel, aerogel, nanofibrous membrane adsorbents, and so on. The chitosan powder with a deacetylation degree of 53% achieved maximum adsorption capacities of 645.1 (0.945), 922.9 (2.040), 973.33 (2.778), 693.2 (1.147), and 728.2 (1.309) mg/g (mmol/g) for Acid Green 25, Acid Orange 10, Acid Orange 12, Acid Red 18, and Acid Red 73, respectively [10]. Dotto et al. reported a chitosan film that exhibited a maximum adsorption capacity of 194.6 mg/g (0.322 mmol/g) for Acid Red 18 [11]. Rêgo et al. prepared a chitosan film using chitosan powder with an 85 ± 1% deacetylation degree, which provided maximum adsorption capacities of 413.8 mg/g (0.774 mmol/g) for Tartrazine and 278.3 mg/g (0.460 mmol/g) for Amaranth [12]. The chitosan nanofibrous membrane obtained a maximum adsorption capacity of 1338 mg/g (1.963 mmol/g) for Acid Blue 113 and an adsorption rate constant (κ) of 9.25 × 10^−5^ g/mg/h [13]. Chitosan hydrogel beads were crafted for removing Congo Red, attaining a maximum adsorption capacity of 223.25 mg/g (0.320 mmol/g) and an adsorption rate constant (κ) of 5.16 × 10^−5^ g/mg/min [14]. A chitosan aerogel was employed as an adsorbent for Direct Yellow 27 removal, demonstrating a rate constant (κ) of 4.35 × 10^−5^ g/mmol/min and a maximum adsorption capacity of 495 mg/g (0.747 mmol/g) [15]. Through the above results, it is found that the maximum adsorption capacities of chitosan with different morphologies and structures are not the same, although those are converted to the number of moles. The adsorption rates and capacities vary under different experimental conditions, making it difficult to determine the most effective morphology and structure of chitosan adsorbents for dye removal.

The adsorption rate may be limited by many factors, such as the size of the adsorbate molecule, the concentration and affinity of the adsorbate to the adsorbent, and the diffusion coefficient of the adsorbate in the bulk phase [16]. In addition, the morphology and structure of the adsorbent should also be considered in relation to the adsorption rate and capacity. Therefore, it is essential to use the same dye and experimental conditions to study how chitosan with different morphologies and structures impacts adsorption rates and capacities. This is of great significance for the design and application of adsorbents. 

In this work, four kinds of chitosan adsorbents with different morphologies were prepared: hydrogel (CSH), aerogel (CSA), powder (CSP), and electrospinning nanofiber (CSEN). CSH, CSA, CSP, and CSEN were characterized using scanning electron microscopy (SEM), X-ray diffraction (XRD), attenuated total reflection Fourier transform infrared spectroscopy (ATR-FTIR), and the Brunauer–Emmett–Teller (BET) method. Under the same experimental conditions, their adsorption behaviors towards Acid Red 27 were analyzed using adsorption kinetics and equilibriums. The relationships between the structures and adsorption properties of the four chitosan adsorbents were discussed to further explore the adsorption mechanisms.

## 2. Materials and Methods

### 2.1. Materials

**Figure 1:**
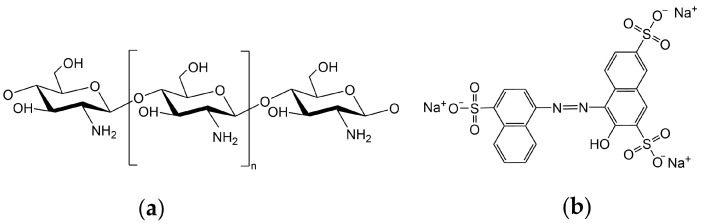
Chemical structures of (**a**) chitosan and (**b**) Acid Red 27 (AR 27).

Chitosan powder (CS, Mw = 1,000,000, 70% deacetylation degree), acetic acid (HAc, AR, 99.5%), sodium hydroxide (NaOH, AR, 97%), 1,1,1,3,3,3-Hexafluoro-2-propanol (HFIP, AR, 99.5%), and Acid Red 27 (AR 27, Mw = 604.5, BS, 85%) were purchased from Shanghai Maclin Co., LTD. The chemical structures of chitosan and AR 27 are shown in Figure 1. The deionized water was used without a special statement.

### 2.2. Preparation of CSP

The purchased chitosan powder was dispersed, and then the pH value of the dispersed solution was adjusted to 5.4 with 0.1% H_2_SO_4_. The sample was washed with deionized water until an approximately neutral pH value was reached. Finally, the sample was dried under vacuum at 60 °C to a constant weight and stored in a desiccator for further use as CSP.

### 2.3. Preparation of CSH

Chitosan hydrogel was prepared by the previously described method [17]. A certain amount of chitosan was dissolved in 1% HAc to prepare 1% chitosan solution. A 10% NaOH solution was dropped into 1% chitosan solution to precipitate chitosan. The precipitate was acidified and washed by the same method that was used for CSP. Finally, the precipitate was centrifuged to remove excess water, and sealed in a centrifugal tube for further use as CSH. A total of 1.120 g of CSH contained 0.020 g dry chitosan (water regain is 0.982 g/g).

### 2.4. Preparation of CSA

CSA was prepared as described by Su et al. [18]. A 1% chitosan solution was frozen and subjected to lyophilization at −70 °C, 1.0 kPa for 36 h. The freeze-dried sample was treated with 1.0% NaOH to neutralize HAc. The freeze-dried sample was acidified and washed according to the method used for CSP. Finally, the sample was dried in vacuum at 60 °C to a constant weight, and stored in a desiccator for further use as CSA.

### 2.5. Preparation of CSEN

A total of 1.0 g of CSH (except for acidification) was dissolved in 35 g of HFIP for 24 h as chitosan electrospinning solution. The spinning voltage, solution feeding rate, needle to collector distance, and temperature were 18.0 kV, 2.0 mL/h, 10.0 cm, and room temperature, respectively. CSEN was acidified and washed by the same method that was used for CSP. Finally, the nanofiber was dried in vacuum at 60 °C to a constant weight to remove residual solvent and placed in a desiccator to further serve as CSEN. 

### 2.6. Characterization

The scanning electron microscopy (SEM, Merlin Compact, Germany Carl Zeiss) method was used to describe the morphologies of CSA, CSP, and CSEN. X-ray diffraction (XRD, D8 ADVANCE, Germany Bruker) analyses of CSH, CSA, CSP, and CSEN were performed to analyze the crystalline structures. The scanning rate was set at 2°/min with an operating voltage of 40 kV and a current of 40 mA. The chemical compositions of the four chitosan adsorbents were analyzed using the attenuated total reflection unit on the Thermo Scientific Nicolet iS50 infrared spectrometer (USA, ATR-FTIR). The specific surface areas of CSH, CSA, CSP, and CSEN were estimated using the Brunauer–Emmett–Teller (BET) analysis (Quantachrome, Autosorb-IQ, USA). The absorbance was measured using a visible-light spectrophotometer (UNICO, 2100, Shanghai, China). Before SEM, BET, and ATR-FTIR testing, CSH was subjected to elution with anhydrous ethanol and then dried using supercritical CO_2_ drying to avoid the disturbance of residual water, while the other three chitosan adsorbents were tested directly.

### 2.7. Adsorption Kinetics

A 500 mg/L AR 27 solution was prepared by diluting 2.0 g/L AR 27 stock solution. A total of 1.120 g CSH (0.020 g dry chitosan), 0.020 g CSA, 0.020 g CSP, and 0.020 g CSEN, respectively, were added into 100 mL of 500 mg/L AR 27 solution. The adsorption experiments were conducted in a shaker at 20 °C with a speed of 150 rpm. The absorbance was determined by taking samples at regular intervals using the spectrophotometer. Each batch adsorption experiment was conducted three times.

The standard working curve of AR 27 was determined as follows. Solutions of AR 27 with initial concentrations of 5, 10, 20, 30, 40, 50, 60, 70, and 80 mg/L were prepared. The absorbances of obtained solutions were measured at the maximum absorption wavelength (λ_max_ = 520 nm). The resulting data were plotted with absorbances as the horizontal coordinate and concentrations as the vertical coordinate. Then, linear fitting was performed, and the fitted equation obtained was the standard working curve of AR 27. 

The standard working curve of AR 27 is as follows:

(1) C=27.9458A−0.7507

where C (mg/L) is the concentration of AR 27 solution and *A* is the absorbance at the wavelength of 520 nm. The linear correlation coefficient R^2^ is 0.9994. The errors of intercept and slope are ±0.3830 and ±0.2564, respectively.

The adsorption capacities of the adsorbent at different times were calculated using the following equation:

(2) $$Q_{t}=\frac{C_{0}-C_{t}}{C_{0}}\times \frac{V}{m}$$

where Q_t_ (mg/g) is the adsorption capacity at time t (min); C_0_ and C_t_ (mg/L) are the dye concentrations at the beginning of the experiment and at time t (min), respectively; V (L) is the volume of solution; and m (g) is the mass of adsorbent.

The adsorption kinetics analysis was based on the pseudo-first-order (PFO) and pseudo-second-order (PSO) kinetic models.

The linear PFO kinetic model can be described by the following equation [19]:

(3) $$\frac{dQ_{t}}{dt}=\kappa_{1}(Q_{e}-Q_{t})$$

Equation (3) can be derived using the following formula:

(4) $$Log(Q_{e}-Q_{t})=LogQ_{e}-\kappa_{1}t$$

The linear PSO kinetic model can be described by the following equation [20]:

(5) $$\frac{dQ_{t}}{dt}=\kappa_{2}{\left(Q_{e}-Q_{t}\right)}^{2}$$

Equation (5) may be expressed in the following form:

(6) $$\frac{t}{Q_{t}}=\frac{1}{\kappa_{2}\times Q_{e}^{2}}+\frac{t}{Q_{e}}$$

where Q_e_ and Q_t_ (mg/g) are the adsorption capacities of adsorbent at equilibrium and time t (min), respectively; κ_1_ and κ_2_ represent the PFO and PSO rate constants, respectively.

### 2.8. Adsorption Isotherm

The experimental AR 27 solutions were prepared by diluting 2.0 g/L stock solution to obtain various concentrations of 10, 20, 30, 40, 50, 100, 200, 300, 500, 750, 1000, and 2000 mg/L. A total of 1.120 g CSH, 0.020 g CSA, 0.020 g CSP, and 0.020 g CSEN were added to 100 mL of AR 27 solutions with different initial concentrations. The adsorption experiments were conducted in a shaker with a speed of 150 rpm at 20 °C, 40 °C, and 60 °C, respectively. Adsorption isotherm analyses were based on the Langmuir and Freundlich models. Each batch adsorption experiment was conducted three times.

The Langmuir isotherm model is expressed as follows [21]:

(7) $$Q_{e}=\frac{Q_{\max}bC_{e}}{1+bC_{e}}$$

Equation (7) can be represented by the following equation [20]:

(8) $$\frac{C_{e}}{Q_{e}}=\frac{1}{Q_{\max}b}+\frac{C_{e}}{Q_{\max}}$$

where Q_e_ (mg/g) is the equilibrium adsorption capacity; C_e_ (mg/L) is the equilibrium concentration; and b is the Langmuir constant. Q_max_ (mg/g) is the maximum adsorption capacity.

The Freundlich isotherm model is expressed as follows [20]:

(9) $$Q_{e}=K_{f}C_{e}^{\frac{1}{n}}$$

Equation (9) can be transformed to the following equation [20]:

(10) $$LogQ_{e}=\frac{1}{n}LogC_{e}+LogK_{f}$$

where K_f_ is the adsorption capacity and 1/n is the strength of adsorption capacity.

## 3. Results

### 3.1. Morphology Analysis

**Figure 2:**
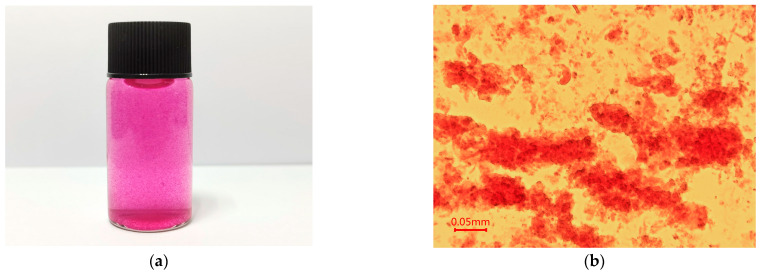

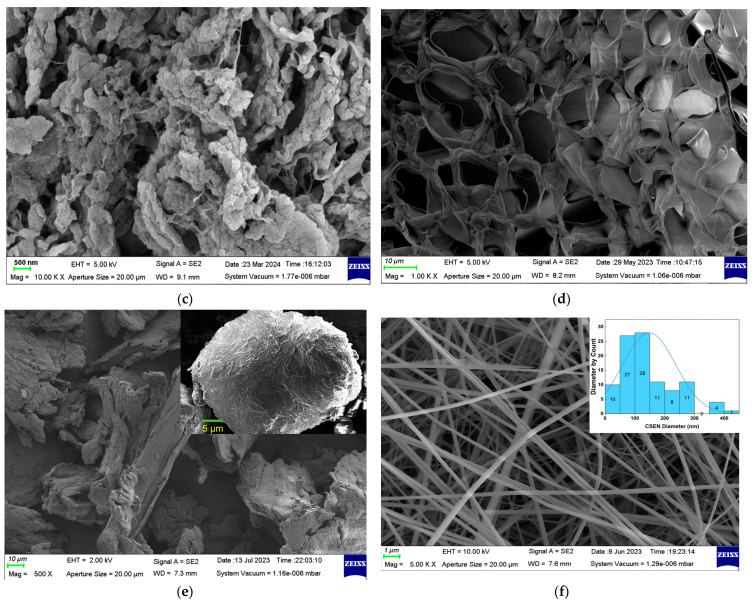
(**a**) Digital image and (**b**) microscope photograph of chitosan hydrogel (CSH) dispersed in AR 27 solution. SEM images of (**c**) CSH, (**d**) chitosan aerogel (CSA), (**e**) chitosan powder (CSP), and (**f**) chitosan electrospinning nanofiber (CSEN).

The surface appearances of CSH, CSA, CSP, and CSEN are shown in Figure 2. CSH appeared to be swollen and flocculent in the AR 27 solution during the adsorption tests, which can be seen from the digital image and microscope photograph in Figure 2a,b. As shown in Figure 2c, the supercritical CO_2_-dried CSH exhibit aggregated fine particles. The sizes of the fine particles are much smaller than CSP. However, in practice, the flocculence of CSH should be significantly smaller than these fine particles. As shown in Figure 2d, CSA possessed a high porosity, smooth surface, and macroscopic 3D network structure, which may be due to the irregular growth of ice crystals and sublimation during the freeze-drying process [22]. CSP had a rough surface, large particle size, and irregular morphology, as observed in Figure 2e. Figure 2f exhibits the clear, smooth, and uniform surface of CSEN. A total of 100 different fibers were randomly chosen for calculating the average diameter of CSEN through accessory SmartTiff V3 software. The diameters of CSEN were between 20 and 420 nm with an average value of 148 nm, which can be detected from the diameter distribution histogram in the top right corner of Figure 2f.

### 3.2. XRD Analysis

**Figure 3:**
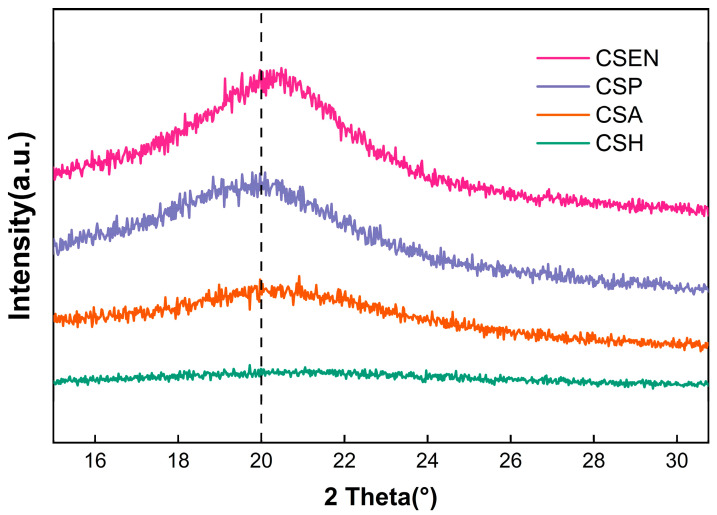
XRD patterns of CSH, CSA, CSP, and CSEN.

XRD patterns showed that the four chitosan adsorbents with different morphologies contained large amounts of amorphous regions and small amounts of semicrystalline structures. As presented in Figure 3, the four chitosan adsorbents had a broad diffraction peak centered at about 2θ = 20°, which was the characteristic feature of semicrystalline domains of chitosan, resulting from the compact arrangement of hydrogen bonds in chitosan [23]. The crystallinities of CSH, CSA, CSP, and CSEN were calculated as 0.41%, 6.97%, 8.76%, and 39.77% using accessory DIFFRAC.EVA software, respectively. The crystallinity of CSH dispersed in the AR 27 solution and may be near zero. The lowest crystallinity of CSH may result from the swelling of the porous network. During the formation of CSH, the disruption of hydrogen bonds between chitosan molecules allowed increased freedom of molecular chain motion, and the water molecules entered among the chitosan molecular chains. Upon freezing, the chitosan solution underwent a volume expansion due to the formation of ice crystals, which led to the arrangement of chitosan molecular chains and an increase in the crystallinity of CSA. The crystallinity of CSP may be attributed to the drying process. CSEN possessed the highest crystallinity, owing to the stretching of chitosan molecules by electrostatic force and volatilization of HFIP from the electrospinning solution, which represented a tight arrangement of chitosan molecular chains.

### 3.3. FTIR Analysis

**Figure 4:**
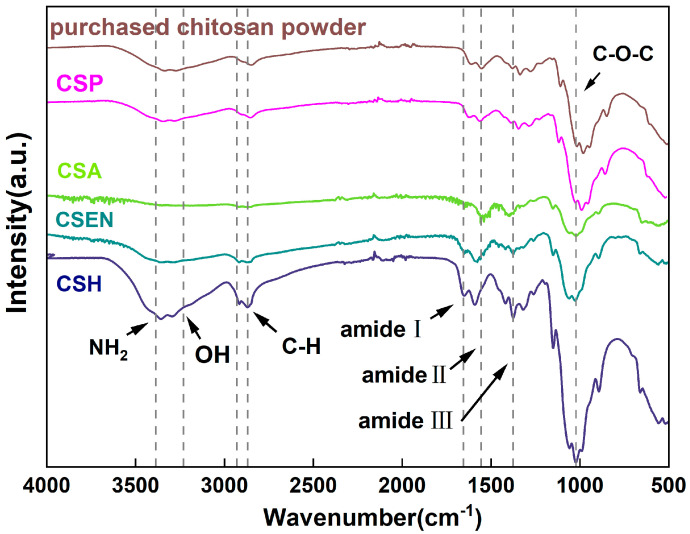
ATR − FTIR spectra of the purchased chitosan powder, CSH, CSA, CSP, and CSEN.

As shown in Figure 4, the typical bands belonging to chitosan were found in the ATR-FTIR spectra of the purchased chitosan powder. The overlapping bands at 3371 cm^−1^ and 3278 cm^−1^ were attributed to the N-H and O-H groups, respectively [24]. The N-acetyl groups were detected in bands at approximately 1656 cm^−1^ (C=O stretching of amide-I) and 1376 cm^−1^ (C-N stretching of amide-III), as well as in the band at 1557 cm^−1^ (N-H bending of amide II). C-O-C stretching was observed at 1150 and 1022 cm^−1^ [25]. The bands of CSH, CSA, CSP, and CSEN were kept the same as the purchased chitosan powder, which indicated that there were no additive residues in the four chitosan adsorbents.

### 3.4. BET Analysis

**Figure 5:**
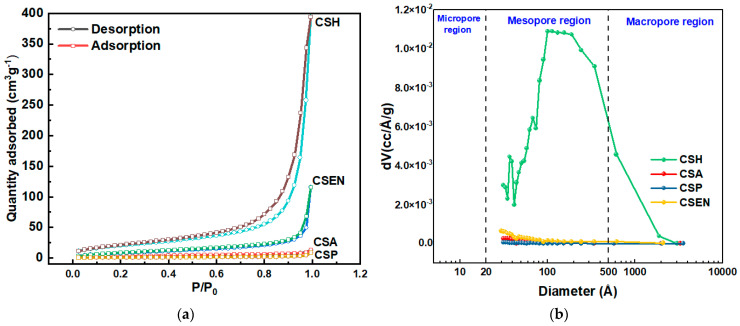
(**a**) N_2_ adsorption–desorption curves and (**b**) pore size distribution of CSH, CSA, CSP, and CSEN. (**a**) the circular symbol denotes the desorption curve, while the square-shaped symbol indicates the adsorption curve.

Figure 5a,b depict the N_2_ adsorption–desorption curves and pore size distribution of the four chitosan adsorbents. According to the IUPAC classification, CSH and CSEN are assigned to a typical type-IV isotherm curve with H1 type hysteresis loops, indicating the presence of mesopores (2–50 nm) in the two adsorbents [26,27]. Figure 5b further indicates that CSH and CSEN possess mesoporous characteristics. The mesopores of CSEN may be caused by the spaces between the nanofibers. In addition, CSA has some pores (>1 μm), which are clearly observed in Figure 2b. The type-II isotherm curves characteristic of CSA, and CSP’s adsorption isotherm, indicate its nonporous nature. The four chitosan adsorbents contain few micropores (<2 nm). The Brunauer–Emmett–Teller (BET) specific surface areas of CSH, CSA, CSP, and CSEN were determined to be 74.6, 9.6, 2.2, and 32.4 m^2^/g, respectively, as presented in Table 1. CSH (after supercritical CO_2_ drying) possesses the highest specific surface area, pore volume, and pore diameter, which cannot fully demonstrate the actual specific surface of CSH during the adsorption tests. In practice, the flocculent CSH should have a huge specific surface area compared to the supercritical CO_2_ dried CSH.

### 3.5. Adsorption Kinetics Analysis

**Figure 6:**
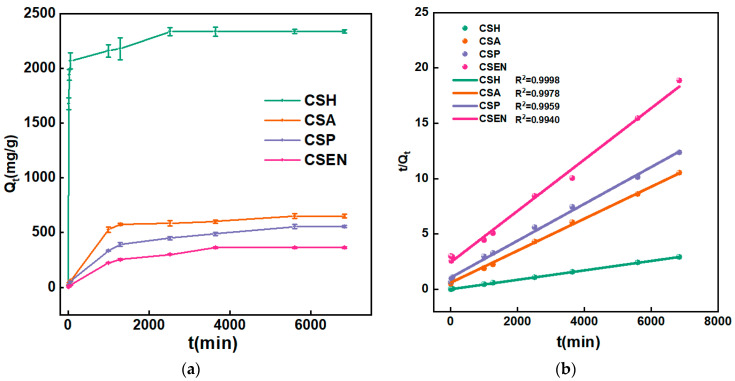
(**a**) Plots of *Q_t_* vs. time for AR 27 on CSH, CSA, CSP, and CSEN. (**b**) Fitting curves of PSO model.

Figure 6a shows the adsorption capacities of CSH, CSA, CSP and CSEN at different times. As time went by, the adsorption capacity of AR 27 gradually increased until it reached equilibrium for each adsorbent. The steeper slope of the plots of *Q_t_* vs. time corresponded to a faster adsorption process. In terms of both rates and capacities of adsorption, the order was CSH > CSA > CSP > CSEN. The plot of *Q_t_* vs. time for AR 27 on CSH looked almost rectangular, which meant that CSH had the fastest adsorption rate. CSH reached over an 88% adsorption capacity within 60 min, while the other three absorbents took almost 1000 min.

The fitting curves for PSO kinetics of CSH, CSA, CSP, and CSEN were carried out as represented in Figure 6b. Table 2 displays the fitting results from the PFO model and the PSO model. Compared to PFO, the PSO model in Table 2 provided a better fitting curve (R_1_^2^ < R_2_^2^) for the adsorption processes of the four chitosan adsorbents for AR 27. The results showed that the adsorption mechanisms of all the four chitosan adsorbents for AR 27 were dominated by chemisorption, which is consistent with previous research reports [27,28,29]. The isoelectric value of the chitosan was reported at pH 6.7 [30], so most of the amino groups were positively charged by protonation below pH 6.7. Electrostatic interaction occurred between the positively charged -NH_3_^+^ of the four chitosan adsorbents and the negatively charged -SO_3_^-^ of AR 27 ions. Generally, the higher κ_2_ value indicates a faster rate of adsorption, resulting in a more rapid attainment of equilibrium. The κ_2_ values suggested that the adsorption rates of the four chitosan adsorbents were in the following order: CSH (1.32 × 10^−5^ mg/g/min) > CSA (3.50 × 10^−6^ mg/g/min) > CSP (2.59 × 10^−6^ mg/g/min) > CSEN (2.18 × 10^−6^ mg/g/min). The κ_2_ values of CSH and CSEN spanned an order of magnitude.

The adsorption mechanism is generally considered to involve three stages: (i) adsorbate mass transfer across the external boundary layer film of liquid surrounding the outside of the adsorbent; (ii) adsorption at a site on the surface of the adsorbent; (iii) diffusion of the adsorbate molecules to an adsorption site, either by a pore diffusion process through the liquid-filled pores, or by a solid surface diffusion mechanism [16]. In this study, the transfer mass rate of dye molecules across the external boundary layer film of liquid surrounding the outside of the four chitosan adsorbents should be similar, owing to the same experimental conditions. The second stage is often assumed to be extremely rapid. The third stage should be the adsorption rate-controlling stage. In other words, the differences in adsorption rates for the four chitosan adsorbents were caused by the diffusion of AR 27 molecules to an adsorption site, either by a pore diffusion process through the liquid-filled pores, or by a solid surface diffusion.

It is commonly believed that chitosan undergoes a glass transition during hydration, marked by the existence of an amorphous region, which facilitates the diffusion of small molecules [31,32]. Hydrogels are an open and accessible matrix, which are able to absorb and retain large volumes of water by the disruption of original hydrogen bonds [31,33]. The hydrogen bonds in the semicrystalline domains of chitosan can hinder the intraparticle diffusion rate of dye molecules. For dye molecules, the diffusion rate through the water-filled pores in hydrogels should be greater than the intraparticle diffusion rate in the amorphous region of the adsorbent, not to mention the semicrystalline domains. Thus, the intraparticle diffusion rate of dye molecules in the bulk phase of the adsorbent may be the main factor limiting the adsorption rate, which is negatively correlated with the corresponding crystallinity. More amorphous regions of adsorbents indicate that more adsorption sites are exposed for dye molecules, as well as the specific surface area. Therefore, CSH with the lowest crystallinity and largest specific surface area has the highest adsorption rate and capacity, while the adsorption rate and capacity of CSEN with the highest crystallinity are the lowest. This can also be confirmed from the adsorption rates and capacities of CSP and CSA. CSEN has a higher specific surface area. Nevertheless, the adsorption rate and capacity of CSEN do not seem to be consistent with its specific surface area, which demonstrates that the crystallinity of the four chitosan adsorbents was the main factor impacting the adsorption rates and capacities, compared with the specific surface area. Compared with CSP, the pores (>1μm) in CSA did not significantly improve its adsorption rate and capacity.

### 3.6. Adsorption Isotherm Analysis

**Figure 7:**
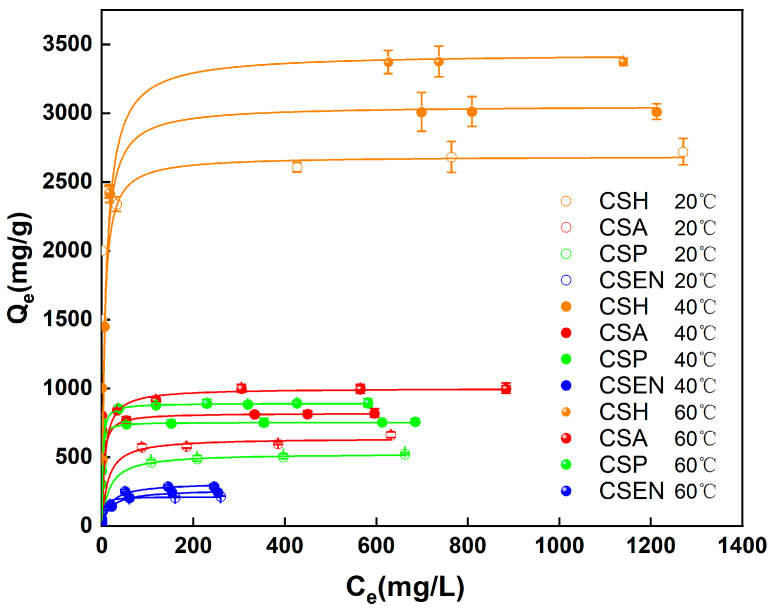
Equilibrium adsorption of AR 27 on the four chitosan adsorbents at different temperatures.

Figure 7 depicts the equilibrium adsorption of CSH, CSA, CSP, and CSEN at 20 °C, 40 °C, and 60 °C. Under identical conditions, the four chitosan adsorbents exhibited different adsorption capacities. The adsorption capacities of each chitosan adsorbent increased as the temperature rose from 20 °C to 60 °C. At different temperatures, CSH has the largest adsorption capacity, while CSEN has the smallest adsorption capacity. But the adsorption capacity of CSA is greater than that of CSP at each temperature. The shapes of all the adsorption isotherms at each temperature are nearly rectangular because the equilibrium adsorption capacities *(Q_e_)* of the four chitosan adsorbents at low equilibrium dye concentrations (*C_e_*) attained almost the same as those at high equilibrium dye concentrations. This demonstrates that all four chitosan adsorbents have high adsorption capacities even at low equilibrium dye concentrations. The rectangular adsorption isotherm curves were also reported in the literature [34].

**Figure 8:**
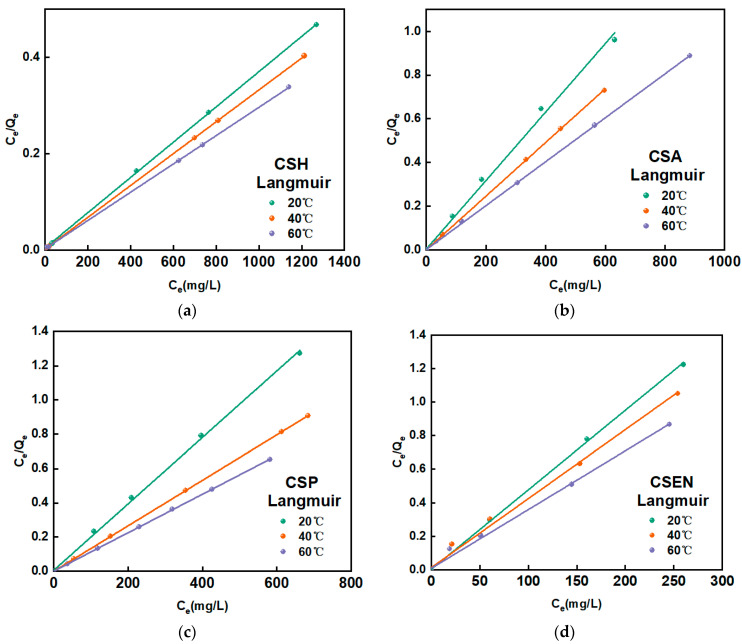
Langmuir plot at different temperatures on (**a**) CSH, (**b**) CSA, (**c**) CSP, and (**d**) CSEN.

The equilibrium adsorption isotherm serves as a fundamental tool for understanding the interaction between the adsorbent and adsorbate. Adsorption isotherms commonly include the Langmuir, Freundlich, Redlich–Peterson, Dubinin–Radushkevich, and BET models, and so on [35]. As most adsorption behaviors for dyes usually follow the Langmuir or Freundlich isotherm models, these two models were employed in this study. The fitting curves of the Langmuir isotherm and the fitting results for both the Langmuir and Freundlich isotherms are shown in Figure 8 and Table 3, respectively. Since R_L_^2^ was higher than R_F_^2^, the Langmuir model was more suitable for describing the adsorption behaviors of AR 27 removal by the four chitosan adsorbents. Therefore, the adsorption behaviors between the four chitosan adsorbents and AR 27 are monolayer adsorption behaviors.

The *Q_max_* values were obtained from the Langmuir isotherm model, and the *Q_max_* values of the four chitosan adsorbents increased gradually at 20 °C, 40 °C, and 60 °C for AR 27. The increases in temperature lead to the more rapid intraparticle diffusion of AR 27. At the same temperature, the *Q_max_* values of the four chitosan adsorbents are ordered as follows: CSH > CSA > CSP > CSEN. This is in accord with the effect of the crystallinities of the four chitosan adsorbents on the adsorption rates for AR 27. More semicrystalline domains of adsorbents indicate that adsorption sites are exposed for dye molecules. At 20 °C, the *Q_max_* of CSH is about 12.7 times that of CSEN. The low crystallinity of CSH cannot only facilitate the intraparticle diffusion of dye molecules, but also the exposure of more adsorption sites.

The *Q_max_* values of the four chitosan adsorbents for AR 27 were much better than the reported non-chitosan adsorbents, as listed in Table 4. The maximum adsorption capacities (mmol/g) of chitosan with different morphologies and structures (powder, beads, films, and nanofibrous membranes) are not the same. It is seen that the *Q_max_* value of CSH is much larger than that of other morphologies of the chitosan adsorbent. After subtracting the undeacetylated amino groups, every gram of the purchased chitosan powder (70% degree of deacetylation) has 4.343 mmol amino groups. Assuming that each amino group in the purchased chitosan powder adsorbed one AR 27 molecule, the theoretical maximum adsorption per gram of chitosan was 5.109 mmol/g for AR 27 (85% purity). The maximum adsorption capacity of CSH achieved 4.523 mmol/g for AR 27 at 20 °C, which is close to the theoretical value. However, the maximum adsorption capacities of the reported chitosan adsorbents are far from the corresponding number of amino groups, which may be owed to the same semicrystalline domains between CSA, CSP, and CSEN. The presence of semicrystalline domains should prevent the amino group from being fully exposed for AR 27 molecules. In semicrystalline domains, the molecular segments of chitosan are closely arranged, resulting in dye molecules which are hard to diffuse. Each molecule of AR 27 contains three sulfonic acid groups. When one sulfonic acid group of AR 27 binds with -NH_3_^+^ of chitosan, the other two sulfonic acid groups may create electrostatic repulsion for subsequent dye molecules. Steric hindrance from electrostatic repulsion may also be one of the factors that impact the maximum adsorption capacity.

## 4. Conclusions

In this work, the adsorption behaviors of the four chitosan adsorbents (CSH, CSA, CSP, and CSEN) with different morphologies and structures for removing AR 27 were comparatively studied in a batch system. The crystallinities of CSH, CSA, CSP, and CSEN were calculated as 0.41%, 6.97%, 8.76%, and 39.77%, respectively. The specific surface areas of CSH, CSA, CSP, and CSEN were measured to be 74.6 (after supercritical CO_2_ drying), 9.6, 2.2, and 32.4 m^2^/g, respectively. The experimental data of the four chitosan adsorbents fitted very well to the PSO, which demonstrated that the adsorption mechanisms of all four chitosan adsorbents for AR 27 were dominated by chemisorption. The adsorption rates of the four chitosan adsorbents are ordered as follows: CSH (1.32 × 10^−5^ mg/g/min) > CSA (3.50 × 10^−6^ mg/g/min) > CSP (2.59 × 10^−6^ mg/g/min) > CSEN (2.18 × 10^−6^ mg/g/min). The Langmuir model was more suitable for describing the adsorption behaviors of AR 27 by the four chitosan adsorbents; these behaviors were indicated as monolayer adsorption behaviors. The maximum adsorption capacities of CSH, CSA, CSP, and CSEN were 2732.2 (4.523), 675.7 (1.119), 534.8 (0.885), and 215.6 (0.357) mg/g (mmol/g) at 20 °C. The crystallinity of the four chitosan adsorbents was the main factor impacting the adsorption rates and adsorption capacities, compared with the specific surface area. With the decrease in crystallinity, the adsorption rates and capacities of the four chitosan adsorbents increase gradually under the same experimental conditions. The maximum adsorption capacity of CSH with the lowest crystallinity and largest specific surface area achieved 3413.0 (5.646) mg/g (mmol/g), which was very close to the theoretical adsorption value and much better than the reported adsorbents.

## Figures and Tables

**Table 1:** Specific surface area (m^2^/g), pore volume (cc/g), and pore diameter (nm) of the four chitosan adsorbents.

Adsorbent	Specific Surface Area (m^2^/g)	Pore Volume (cc/g)	Pore Diameter (nm)
CSH	74.6	0.62	11.34
CSA	9.6	0.02	3.5
CSP	2.2	0.01	3.3
CSEN	32.4	0.17	3.0

**Table 2:** CSH, CSA, CSP, and CSEN adsorption parameters of PFO and PSO model (Temperature = 20 °C).

Adsorbent	PFO			PSO		
	κ_1_ (min^−1^)	*Q_e_* (mg/g)	R_1_^2^	κ_2_ (mg/g/min)	*Q_e_* (mg/g)	R_2_^2^
CSH	1.22 × 10^−2^	680.6	0.5016	1.32 × 10^−5^	2346.9	0.9998
CSA	7.66 × 10^−3^	551.0	0.8190	3.50 × 10^−6^	689.7	0.9978
CSP	5.92 × 10^−4^	540.4	0.9548	2.59 × 10^−6^	602.4	0.9959
CSEN	7.42 × 10^−4^	221.7	0.9656	2.18 × 10^−6^	431.0	0.9940

**Table 3:** Adsorption isotherm constants for AR 27 on CSH, CSA, CSP, and CSEN.

Adsorbent	T (°C)	Langmuir Isotherm			Freundlich Isotherm		
		*Q_max_* (mg/g)	b	R_L_^2^	*K_f_* (mg/g)	1/n	R_F_^2^
CSH	20	2732.2	0.005	0.9999	2.255	0.029	0.9981
	40	3030.3	0.002	0.9999	2.069	0.135	0.9864
	60	3413.0	0.003	0.9999	2.258	0.085	0.9841
CSA	20	675.7	0.042	0.9954	2.534	0.065	0.7729
	40	819.7	0.006	0.9999	2.852	0.009	0.9928
	60	1005.0	0.008	0.9999	2.907	0.016	0.9128
CSP	20	534.8	0.038	0.9997	2.618	0.066	0.9932
	40	757.6	0.003	0.9999	2.838	0.027	0.9772
	60	892.9	0.002	0.9999	2.844	0.056	0.8725
CSEN	20	215.5	0.023	0.9995	3.305	0.042	0.8463
	40	260.4	0.064	0.9988	3.314	0.056	0.8719
	60	303.0	0.046	0.9981	3.288	0.082	0.8830

**Table 4:** Comparison of *Q_max_* values of adsorbents for some dyes.

Adsorbent (Deacetylation Degree)	Dye	*Q_max_* (mmol/g)	NAG * (mmol/g)	Reference
CSH (70%)	AR 27(85% purity)	4.523	4.343	This study
CSA (70%)		1.119		
CSP (70%)		0.885		
CSEN (70%)		0.357		
Chitosan powder (53%)	Acid Green 25	0.945	3.289	[10]
	Acid Orange 10	2.040		
	Acid Orange 12	2.778		
	Acid Orange 18	1.147		
	Acid Red 73	1.309		
Chitosan film (85 ± 1%)	Acid Red 18	0.322	5.274	[11]
Chitosan film (85 ± 1%)	Tartrazine	0.774	5.274	[12]
	Amaranth	0.460		
Chitosan nanofibrous membrane (≥95%)	Acid Blue 113	1.963	5.894	[13]
Chitosan beads(cross-linked) (85.5%)	Reactive Red 189	1.704	5.305	[36]
Chitosan beads(non-cross-linked) (85.5%)		1.046		
Composted pine bark	AR 27	0.007	-	[37]
Municipal solid waste compost		0.117		
Water hyacinth leaves		0.117		[38]
Fe_3_O_4_/MgO/ nanoparticles		0.063		[39]
MgAlCO_3_		0.200		[40]
CS-PEI-GLA		0.080		[41]

* NAG: The mole number of amino groups of per gram chitosan adsorbent. NAG= 1 ÷ 161.2 × DD; 161.2 is the molecular weight of the chitosan repeating units; DD is the deacetylation degree of chitosan adsorbent.

## Data Availability

Data are contained within the article.
